# Uncover the trends, gaps, and main topics on online grocery shopping: Bibliometric analysis

**DOI:** 10.1016/j.heliyon.2024.e25857

**Published:** 2024-02-12

**Authors:** Terrylina A. Monoarfa, Ujang Sumarwan, Arif Imam Suroso, Ririn Wulandari

**Affiliations:** a Department of Digital Business, Faculty of Economics, Universitas Negeri Jakarta, Jakarta, 13220, Indonesia; b School of Business, IPB University, Bogor, 16680, Indonesia; c Faculty of Economics and Business, Universitas Mercu Buana, Jakarta, 11650, Indonesia

**Keywords:** Online grocery shopping, Bibliometric analysis, Systematic literature review

## Abstract

This study uses the Preferred Reporting Items for Systematic Review and Meta-Analysis (PRISMA) approach to curate many papers and select documents according to specific qualifications to include in a systematic literature review. At the identification stage, there are 1221 abstract documents showing their relation to online grocery shopping. Furthermore, the researcher selected 571 Scopus abstracts at the screening stage, and then through the VOS Viewers software, the researchers conducted a bibliometric analysis. The bibliometric analysis in this study aims to find trending topics and research gaps in online grocery shopping. At the eligibility stage, there are 170 full-text articles related to the issue of online grocery shopping; until the last stage, through manual selection, the researcher determined 43 full-text articles that met the qualifications for in-depth analysis using a systematic literature review approach. The research findings state that there are several main topics on online grocery shopping, such as customer segmentation and shopping preferences, grocery online shopping behavior, and intentions on online grocery shopping. The findings of this study explain the factors that influence the decision to use online grocery shopping that become a reference for further research to design a model of online grocery shopping intentions.

## 1 Introduction

Retailing, in general, is an activity of buying and selling goods and services. A retail store exists to provide sales of goods and services to end-consumers. Quality of service is an essential factor that affects the high intention to shop. In this case, service quality includes a shopping atmosphere and ease of access to retail store locations [1], in line with Parasuraman et al. service quality includes physical service quality, reliability, responsiveness, empathy, and service assurance [2]. The better the service quality, the more likely it is to increase customer satisfaction, which encourages customer loyalty, as indicated by the high frequency of shopping and the number of items purchased [3]. The retail industry has made physical and service innovations to enhance customer satisfaction and achieve business sustainability [4,5]. Physically, the retail store that used to be a department store has changed its format to a supermarket with a self-service concept that offers many price promotions. Then, over time, supermarkets have developed into supercenters that provide a complete and comprehensive range of personal and household needs.

Meanwhile, based on promotional and marketing media, the retail business was initially promoted through catalogs and then electronically via telephone and TV. In fact, along with the development of internet technology, recently, retail stores have websites and e-commerce platforms to enhance electronic marketing services to customers further. Based on the analysis presented by Pantano [5], one of the rapidly growing retail industries is the grocery sector.

Frank defines *grocery retail* as a retail store that sells daily consumer goods. Regarding product storage and display arrangements, groceries are divided into two primary classifications: dry and wet [6]. Dry grocery is synonymous with more durable products, such as hygiene and personal care products, care products and household supplies, oils and sauces, and dry processed foods (such as cereal, pasta, snacks, or canned food). Meanwhile, wet grocery consists of products that require special treatment to store or display because these products tend to be perishable. Several wet grocery products are perishable, including fresh/frozen protein, vegetables, fruits, dairy products, and frozen processed foods. Frank et al. [7] stated that the factors influencing the intention to shop for groceries include socio-economic factors, prices of goods, availability, and shopping habits. Furthermore, Frank said socio-economics is the most influential in a person's shopping basket size [7]. The socio-economic factors include the number of adults and children in the family, income, education, and employment. The high consumption level of some vegetable commodities in the community also demanded relatively high availability in the market [8].

Based on the general description of the e-grocery retail business that has been presented, this study wishes to present a bibliometric analysis that examines previous research on the retail grocery business and describes the transformation journey of the retail grocery industry from year to year across countries, as well as what challenges need to be resolved as a direction in a future research agenda. Specifically, this research aims to.

(1) Analyze research trends of online grocery shopping during 2012–2022.
(2) Analyze research gaps in online grocery shopping.
(3) Discuss and analyze the primary issues and challenges of online grocery retail industry.

In addition, to synthesizing the body of knowledge already in existence, this literature review and bibliometric analysis offers a path forward for future studies in the online grocery retail space. The result can be used by scholars, business experts, and legislators to inform decision -making, develop plans of action, and deal with the evolving online grocery landscape.

## 2 Literature review

Technological advancements have been instrumental in the evolution of the grocery retail sector. According to Rivera et al. [9] technological developments bringing novelty in carrying out business processes aimed at supply chain optimization, reducing costs, and improving the quality of customer service. Based on technological developments, the evolution of grocery retail underwent several phases, namely: 1) 1980 – barcode and scanning technologies, 2) 1996 – electronic devices, 3) 2001 – internet grocery, 4) 2003 – Radio Frequency Identification (RFID), 5) 2005 – self-service technology (self-serve checkout), 6) 2009 – grocery mobile application, and 7) 2016 – Internet of things. Evolution in grocery retail occurred because modern society demands innovation that simplifies processes and activities. Grocery services oriented toward customer convenience and saving shopping time are the targets of the new e-grocery value proposition [10]. It aligns with the business model statement of *“how companies create and deliver value to customers and then turn the payments received into profits”* [11]. As Darley et al. [12] have developed the Engel-Kollat-Blackwell (EKB) decision-making model into the online purchasing concept, which states that several external factors support online purchasing decisions, including individual characteristics, social influences, situational and economic factors, and online environment. Application of a traceability system has already been implemented to ensure the safety issue for wet products [13]. The internet is a leading factor in increasing the agriculture value-added in several regions [14], implicating the higher purchasing of wet groceries like vegetables or fruits.

2013, Indonesia's e-grocery market grew due to several factors, including changing consumer preferences, convenience, personalization, flexibility, variety, cost savings, and safety [15]. The online grocery sector provides a time-saving alternative for busy urban consumers who want easy access to a diverse choice of products. The Indonesian online grocery market demonstrates significant geographical variances and dynamics, with the online grocery business proliferating in urban areas such as Jakarta, Surabaya, and Bandung, where there is a more considerable proportion of tech-savvy consumers and superior logistics. In a meta-analytical review, Tyrväinen & Karjaluoto identified key determinants influencing online grocery shopping include perceived ease-of-use, usefulness, risk, trust, price value, and social influence [16]. Previous studies have delved into the multifaceted aspects of online grocery shopping, by employing diverse theoretical frameworks. From a technological standpoint, researchers have utilized model such as the technology acceptance model (TAM) and the unified theory of acceptance and use of technology (UTAUT). Psychological perspectives, stemming from the theory of planned behavior (TPB), have also been employed to explore the factors impacting online grocery shopping. The sophisticated understanding of the driving forces behind the growth of Indonesia's e-grocery market underscores the complex interplay of technological, psychological, and societal factors influencing consumer behavior in the rapidly evolving digital landscape.

The booming online retail business in Indonesia has encouraged the government to take policies to improve e-commerce governance in Indonesia. In 2020, the E-commerce Law was issued regarding guidelines for domestic and foreign e-commerce entities and provisions for establishing representative offices in Indonesia. These regulations impact e-grocery by establishing legal standards and compliance requirements. Additionally, compliance with food safety regulations is crucial for e-grocers to ensure product safety [17]. Various e-grocery applications in Indonesia are innovating and competing, including Tanihub, Sayurbox, Segari, HappyFresh, GoMart, GrabMart, AlfaCart, and Klik Indomaret, has stimulated consumer curiosity to try online grocery shopping services [15]. Overall, the projection of the e-grocery market in Indonesia is estimated to grow by 24.30 percent (2023–2027), resulting in a market volume of US$23.87 billion in 2027 [18].

Numerous challenges confront the online grocery retail sector, notably the geographic diversity and complex logistics infrastructure within the country [19]. Despite these challenges, the COVID-19 pandemic has accelerated the growth of the e-grocery market in Indonesia, and more and more businesses in Indonesia are starting to offer e-grocery services [20,21]. Referring to the online purchasing decision process, the COVID-19 pandemic from 2020 to 2021 was a situational and economic factor that proved to be one of the causes of the high online grocery shopping transactions at that time [12]. The mobility restriction policy during the pandemic caused every family to prefer to avoid eating at restaurants to reduce the risk of virus transmission and buy groceries online, then cook them themselves at home [22]. Furthermore, the limited stock availability at grocery stores at that time had created anxiety, so people tended to make large quantities to anticipate shortages of food stocks during self-isolation [20]. Of the various reasons for online grocery shopping during the pandemic, online grocery shopping transactions in Indonesia have increased by 30 percent [23]. Additionally, Gomes & Lopes posited that socio-demographic factors played a pivotal role in influencing the intention to engage in online grocery shopping during the pandemic [24]. Variables such as gender, age, academic qualification, annual income, and consumer behavior exerted a discernible impact on the online grocery experience, ultimately influencing the composition of the shopping basket. This nuanced understanding sheds light on the multifaceted dynamics of online grocery consumption, particularly during a period characterized by unprecedented global challenges.

Outside of a pandemic, online grocery shopping services are an alternative and complement [25]. Customers can choose physical or online grocery shopping services that suit their preferences, lifestyle, household conditions, or busy work schedules. Various studies mention several factors influencing the intention to shop online for groceries. Droogenbroeck & Van Hove through the concept of the Unified Theory of Adopt and Use Technology (UTAUT), stated that the factors of quality technology, facilities, price values, hedonic motives, and social influence behavioral intentions to use e-grocery services [27]. Then, adding personal innovation factors, risk perception, shopping convenience, and service quality are also considered factors driving behavioral intention to use e-grocery services. In line with Droogenbroeck & Van Hove [27], Zehir & Narcıkara also mentioned the importance of e-service quality and e-recovery service quality as drivers of perceived value and intention to be loyal [28]. In addition, Nguyen et al. stated that order fulfilment, including inventory management, last-mile delivery, and returns management, is a driving factor for the behavioral intention in the grocery retail online business [10]. Even García et al. argue that delivery quality is one of the value propositions of e-grocery shopping services [29].

## 3 Methodology

**Fig. 1:**
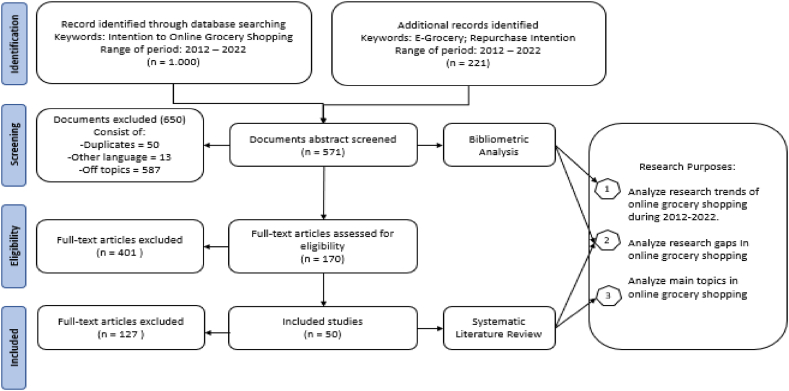
Prisma flow diagram.

An international group of researchers and methodologists created the PRISMA diagram in 2009 as a part of the Preferred Reporting Items for Systematic Review and Meta-Analysis. PRISMA flow diagram is a method of selection, and the article process contains four stages: identification, screening, eligibility, and include. The PRISMA guidelines were developed to increase the transparency and completeness of reporting in systematic reviews, consequently enhancing the reliability and accuracy of the evidence synthesized in these sorts of studies. The PRISMA flow diagram, in particular, became a frequently employed instrument for depicting the flow of information throughout the systematic review process. This gradual selection eliminates many datasets, so a systematic literature review only aims at selected documents that are genuinely on the topic [30,31]. Fig. 1 illustrates the steps for filtering article documents using the PRISMA systematics.

The general stages of PRISMA systematics are.

1. Identification stage

In the preliminary phase, the researchers identified articles using the Scopus database, employing Boolean operators “OR” and “AND” to narrow the search. Documents containing either “Online grocery shopping” OR “E-grocery” were retrieved, with the Boolean operators being employed utilizing to craft nuanced and exact search queries, assuring adaptability and accuracy in information retrieval. Furthermore, the authors used the Boolean operator “AND” to focus on papers specifically related to “Intention to online grocery shopping AND repurchase intention to e-grocery.” The information offered in the introduction section, highlighting the emergence of e-grocery development in Indonesia around 2013, influenced the selection of the precise time period. The researchers aimed to capture the evolution over a ten-year period, providing insights into the current situation of e-grocery and anticipating future directions in the field.

2. Screening stage

Transitioning to the second stage, the screening process involved meticulously selecting documents by eliminating duplicates, non-English articles, and those beyond the study's scope. During this screening phase, 650 documents were excluded, and a total of 571 documents were deemed relevant based on their titles and abstract content, aligning with the research topic. The author employed the Mendeley reference manager to conduct this screening, with a specific focus on titles and abstracts that delved into the specific topics related to factors influencing the behavioral intention to participate in online grocery shopping.

Furthermore, the selected 571 abstract documents were saved in RIS data format as a bibliographic dataset compatible to be calculated and analyzed on the bibliometric analysis process. Bibliometric analysis is an analytical method that aims to summarize large amounts of bibliographic data and visualize it in an intellectual structure so that a trend is drawn in a particular scope of the study [32]. The researcher considers bibliometric analysis because the scope of research on grocery retail is comprehensive. In addition, the number of documents recorded was 571 abstracts, making it impossible to perform manual analysis. To examine large datasets, the researcher conducted a bibliometric analysis with the help of VOS Viewer 1.6.19 software.

The researcher opted for VOS Viewer to undertake bibliometric analysis for various reasons. The software's strong visualization capabilities are noteworthy since it excels at creating intelligent visual representations, notably term maps. Its capability for extensive analysis is critical, allowing for in-depth investigation of bibliometric data. Additionally, the integration capabilities of VOS Viewer, allowing seamless interfaces with other databases sources and rapid data imports, were pivotal in the selection process. The commitment to regular updates by the developers further solidified the choice, ensuring that the software consistently incorporates the latest features and enhancements in the field of bibliometric analysis.

The outcome of the calculations and analysis performed on the VOS Viewer dataset yields a visual representation of trends. The researcher's analysis of these trends provides an insightful overview of the evolutionary trajectory of grocery retail trends over the years. Changes in trends over time serve as indicators of emerging phenomena. Researchers can leverage the results of the bibliometric visualization to establish the novelty of research, identifying gaps that previous studies have yet to address. This bibliometric analysis specifically addresses the first research objective, aiming to scrutinize trends in grocery retail development across various countries from 2012 to 2022.

3. Eligibility stage

Subsequent to the bibliographic analysis of multiple abstracts, the researchers proceed to the PRISMA selection stage to address the second and third research objectives. In the third stage, document selection is contingent upon eligibility criteria. During this phase, 401 articles are excluded, while 170 are deemed eligible. Eligibility is gauged based on the journal's reputation and the accessibility of the journal as an open-access platform, facilitating researchers' access to full-text articles.

4. Included stage

Moving to the final stage, out of the 170 full-text articles, a refined selection on 50 articles is made manually, deemed to possess comprehensive alignment with the research topic aimed at answering the research objectives. These selected articles will undergo systematic examination in the forthcoming literature review.

A systematic literature review is an analytical method that aims to summarize and synthesize the findings put forward by the literature to become material for study and discussion for further research on specific topics [32]. The systematic literature review was chosen as a method of analysis on a limited number of specific topics, making it possible for researchers to conduct manual reviews. Furthermore, Table 1 explains the distribution of published articles about online grocery shopping in Scopus-indexed journals and publishers.

**Table 1:** Papers distribution based on Scopus -indexed journals and publisher.

Publisher	Journal	Number of papers
Elsevier	Australasian Marketing Journal	1
	J. of Retailing and Consumer Services	5
	Technological Forecasting and Social Change	1
	J. of Transport Geography	1
	Research in Transportation Economics	3
	J. of Cleaner Production	1
Emerald	Marketing Intelligence & Planning	1
	J. of Indian Business Research	1
	Business Process Management Journal	1
	Int. J. of Physical Distribution & Logistic	1
	Int. J. of Retail & Distribution Management	3
	British Food Journal	2
	European Journal of Marketing	1
	China Agricultural Economic Review	1
Informs	Marketing Science	1
MDPI	Behavioral Sciences	1
	Sustainability	4
	J. of Open Innovation	1
Routledge	The Service Industries Journal	1
	The Int. Review of Retail, Distribution and Consumer Research	1
	J. of Int. Consumer Marketing	1
	J. of Int. Food and Agribusiness Marketing	3
	J. of Internet Commerce	1
	J. of Food Products Marketing	2
	The Chinese Economy	1
Sage	Vision	1
	Business Perspective and Research	1
	Global Business Review	1
	Metamorphosis	1
Science Direct	J. of Interactive Marketing	1
Springer	European Retail Research	1
Taylor & Francis	Int.l J.l of Logistics Research and Applications	1
	Int. J. of Human–Computer Interaction	1
Wiley	J. of Consumer Behavior	1
	Int. J. of Consumer Studies	1
**Numbers of papers reviewed**		**50**

Based on Tables 1 and it can be seen that among the 50 articles published from 2012 to 2022, Elsevier is the publisher that has published the most papers on online grocery shopping from 2012 to 2022, namely 12 papers, even the Journal of Retailing and Consumer Service has made the most significant contribution by has published five articles. The Journal of Research in Transport Economics has published three articles. Furthermore, Emerald is also a publisher that has published many papers on online grocery shopping in the last ten years. The International Journal of Retail and Distribution Management has made a sizable contribution: three out of 11 published articles. In addition, Routledge has also published many papers on online grocery shopping; even the Journal of International Food and Agribusiness Marketing has contributed to publishing three of the ten articles published by Routledge. Apart from that, MDPI has also published six articles on online grocery shopping and four of them have been published by the Journal of Sustainability. Furthermore, Sage, Wiley, Taylor & Francis, Science Direct, Springer, and Informs published several online grocery shopping articles from 2012 to 2022. Overall, it can be said that online grocery shopping is an exciting topic for researchers to explore.

## 4 Results and discussion

### 4.1 Bibliometric analysis

Upon conducting a keyword search using “intention to online grocery shopping AND repurchase intention to e-grocery,” a total of 521 full-text articles were identified. Subsequently, bibliometric analysis using VOS Viewer involved several key steps.

**Fig. 2:**
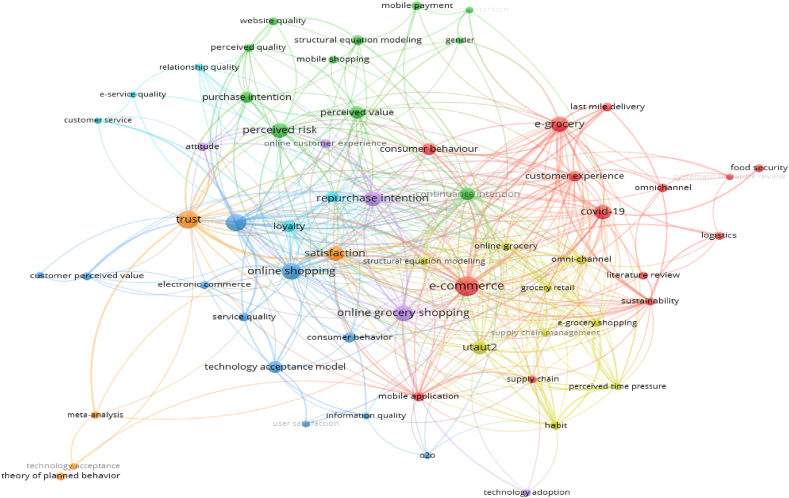
Network visualization of data.

1. Data import – the analysis commenced with data import, utilizing the RIS file type, containing information from 521 identified documents
2. Visualization type – the visualization type chosen was based on co-occurrence with keywords, with keywords serving as the analysis unit. The full-counting calculation method was employed.
3. Parameter adjustment – parameters were fine-tune by establishing thresholds for connections, determining the size and color of nodes, and specifying criteria for inclusion or exclusion. Notably, the researcher set a minimum keyword occurrence of 7 out of 1527 existing keywords.
4. Threshold setting and network generation – the minimum keyword occurrence threshold resulted in 60 items meeting the criteria, forming 443 total links with a cumulative strength of 687. The network was then generated based on these parameters.
5. Visualization presentation – VOS Viewer created a visual representation of the relationships between entities in the bibliometric data, encapsulating the interconnections within the 521 full-text articles.
6. Analysis of clusters and patterns – post-visualization, the analysis delved into clusters and patterns within the network. Each node represented a keyword entity from an article, and clusters of nodes suggested thematic or disciplinary connections.
7. Key analysis points – noteworthy aspects in the visualization included:
  a. Node size indicating the frequency of keyword occurrences (co-occurrences).
  b. The relationship between nodes revealing linkages between keywords.
  c. Link thickness denoting the frequency of co-occurrences between keywords.
  d. Larger the node representing higher occurrences of keywords.
  e. Ticker links indicating increased co-occurrence frequency between keywords.
8. Visual representation – the VOS Viewer present several visualizations include network, overlay, and density visualization. Fig. 2 encapsulates the visualization of the calculations and VOS Viewer analysis of the 521 full-text articles.

In a bibliometric study, network visualization used to analyze relationship between various keywords occurred on the full-text articles. For instance, in keywords co-occurrence networks, nodes represent keywords, and edges signify the co-occurrence of keywords in the same document. This visual representation aids researcher in uncovering patterns of trends and clusters within a vast corpus of literature.

Based on the visualization presented in Fig. 2, from 2012 to 2022, there will be seven clusters (groups of topics) with co-occurrence strength (network strength with other keywords). The topics that are described as having high co-occurrence strength are.

• Cluster 1 (14 items): consumer behavior, COVID-19, customer experience, e-commerce, e-grocery, food security, last mile delivery, literature review, logistics, mobile application, omnichannel, supply chain, sustainability, systematic literature review.
• Cluster 2 (11 items): continuance intention, gender, mobile payment, mobile shopping, perceived quality, perceived risk, perceived value, purchase intention, structural equation modeling, switching intention, website quality.
• Cluster 3 (11 items): consumer behavior, customer perceived value, customer satisfaction, electronic commerce, fresh food e-commerce, information quality, o2o, online shopping, service quality, technology acceptance, user satisfaction.
• Cluster 4 (9 items): grocery shopping, grocery retail, habit, omni-channel, online grocery, perceived time pressure, structural equation modeling, supply chain man-agement, UTAUT2.
• Cluster 5 (5 items): attitude, online customer experience, online grocery shopping, repurchase intention, technology adoption.
• Cluster 6 (5 items): customer loyalty, customer service, e-service quality, loyalty, relationship quality
• Cluster 7 (5 items): meta-analysis, satisfaction, technology acceptance, theory if planned behavior, trust.

**Fig. 3:**
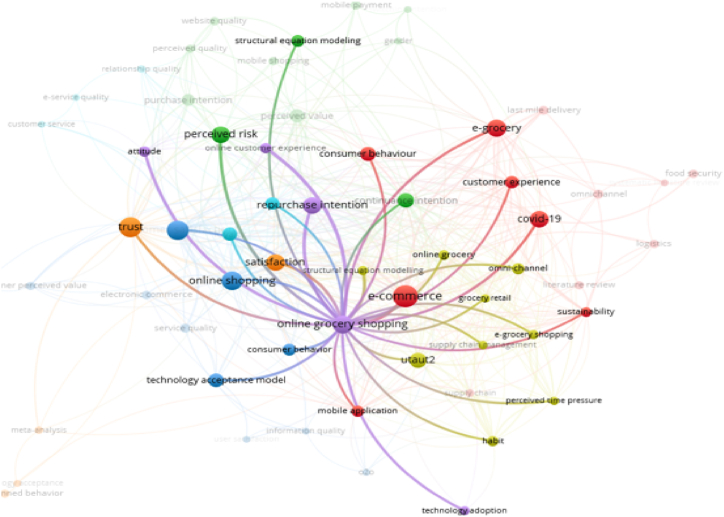
Cluster of papers on online grocery shopping from 2012 to 2022.

Furthermore, based on the visualization of inter-node relationships that describe the linkages between keywords, the topic of online grocery shopping is connected to 7 clusters that presented in Fig. 3 below.

According to Fig. 3, the visualization explains that the topic of online grocery shopping from 2012 to 2022 is often connected to research topics about e-commerce and online shopping. Several studies have utilized concepts such as consumer behavior, the technology acceptance model, and UTAUT2 to explore topics related to satisfaction, repurchase intention, continuance intention, trust, perceived risk, customer experience, COVID-19, attitude, omni-channel, and sustainability within the scope of online grocery shopping or e-grocery. It is even evident that the structural equation modeling analysis method frequently appears as a keyword in studies on online grocery shopping.

**Fig. 4:**
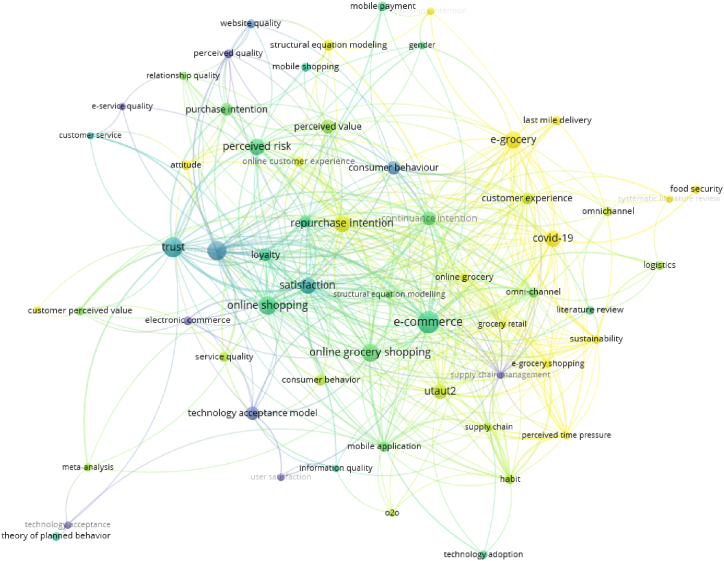
Overlay visualization of data.

VOS Viewer also presented overlay visualization involved the integration of numerous layers of information onto a single network graph. The layers (overlays) indicate distinct dimensions of data. Researcher can acquire a more comprehensive understanding of the relationships and trends within a scholarly landscape by merging multiple data sets in one graphic Overlay visualization in bibliometric analysis allows researcher to investigate major topics links and the temporal evolution of research. Fig. 4 illustrates the overlay visualization of the distribution of research topics during 2012–2022.

Based on the overlay visualization, Table 2 showed the topic of online grocery shopping during 2012–2022.

**Table 2:** Topic of online grocery shopping during 2012–2022.

2012–2015	2016–2017	2018–2019	2020–2022
1. Technology acceptance model	1. Consumer behavior	1. Continuance intention	1. E-grocery
2. Website quality	2. Trust	2. Purchase intention	2. COVID-19
3. Perceived quality	3. Satisfaction	3. Perceived value	3. Repurchase intention
4. E-service quality	4. Loyalty	4. Omni-channel	4. Attitude
5. User satisfaction	5. E-commerce	5. Logistics	5. Last-mile delivery
6. E-commerce	6. Online shopping	6. Mobile application	6. Food security
7. Supply chain management	7. Online grocery shopping	7. UTAUT2	7. Perceived time pressure
	8. Perceived risk	8. Consumer behavior	8. Online grocery
	9. Service quality	9. Habit	9. E-grocery shopping
	10. Theory of planned behavior	10. Customer experience	10. Sustainability
	11. Information quality	11. Service quality	11. Switching intention
	12. Mobile shopping	12. Supply chain	12. Systematic literature review
	13. Mobile payment	13. Meta-analysis	
		14. Structure equation modeling	

### 4.2 Research trends

According to information on Table 2, from 2012 to 2015, research on online grocery shopping discussed the application of the Technology Acceptance Model to predict the intention to adopt online grocery shopping services. Venkatesh et al. Choi, and Groβ expand on TAM and state that the determinants of repurchasing behavior in online grocery shopping services are perceived ease of use and perceived usefulness and perceived risk, social influence, and shopping enjoyment as drivers of satisfaction [[33], [34], [35]]. In addition, Martinelli & Balboni (2012) argue that perceived service quality also contributes to increasing satisfaction to increase customer loyalty [3]. In online grocery shopping services, the quality of electronic services on websites and saving shopping time fostered shopping intentions [36]. In line with Zhu and Semeijn, Gao et al. stated that system and information quality can increase user satisfaction and trust in online shopping applications [37]. The technological performance on websites and mobile applications can moderate performance value to perceived value, indirectly increasing user satisfaction and loyalty [38]. At the same time, Chintagunta et al. focused more on the importance of supply chain management in grocery retail, including setting delivery times and transaction costs [39]. Furthermore, Subramanian et al. added that logistics services increase the competitiveness of online retail businesses [40].

From 2016 to 2017, research trends focused more on maintaining a retail grocery business. In terms of consumer behavior, online grocery shopping customers tend to have practical and modern lifestyle characteristics, which influence their perception of shopping value [41]. Van Droogenbroeck & Van Hove stated that socio-demographic characteristics impact the intention to adopt online grocery shopping – that the factors of household customers of online shopping services at the supermarket in Belgium are young household with young children [42]. One of the motivations for online grocery shopping is the situation of household chores due to the presence of small children - so consumers need solutions to make their household affairs easier. Next, reviewing strategies to increase customer satisfaction, trust, and loyalty, Anesbury et al. stated that the ease of using shopping applications and saving shopping time is one of the attractions for new customers to shop for groceries online [43]. Likewise, Alavion et al. through a theory of planned behavior explained that technology should offer convenience to its users, just as digital marketing applications make it easier for users to make transactions and provide efficiency in the transaction process [44] – so that these services can encourage user motivation and self-efficacy. Thus, the research results provide input to managers of online grocery shopping services to provide practical shopping applications and make it easier for new users to adapt to using them. Efforts to modernize the management of the retail grocery business are, of course, not only to improve service quality and shopping experience but the modernization of logistics services also aims to improve product quality and food freshness [45,46]. As Moriuchi & Takahashi stated, promotional activities, pricing, and improving the shopping experience are important factors that can increase customer satisfaction and potentially foster repurchase intentions [47]. Regarding product quality, Maggioni stated that product freshness, product diversity, and product availability are factors driving loyalty [48]. In addition, Mortimer et al. argue that perceived risk and trust are predictors of customer loyalty [49].

From 2018 to 2019, research trends regarding online grocery shopping have integrated models to obtain a more comprehensive study of the factors influencing repurchase intention for online grocery shopping services. De Toni et al. and Jilcott Pitts et al. stated that in addition to user characteristic factors, healthy food, perceived price fairness, and perceived quality are also factors that influence the perceived value of online grocery shopping, which in turn contributes to encouraging repurchase intentions [50,51]. Likewise, e-service quality and food quality influence perceived value which drives customer loyalty using food delivery services [52]. Furthermore, Kaswengi & Lambey-Checchin added the importance of logistics service quality and product quality to increase customer satisfaction which is indicated by an increase in the number of purchases and the frequency of shopping in the future [53]. In addition, Vakulenko et al. argue that customer experience and the quality of delivery services can increase user satisfaction with online grocery shopping services [54]. Next, Loketkrawee & Bhatiasevi have extended the technology acceptance model by adding the potential for convenience and entertainment in influencing online grocery shopping intentions [55]. Meanwhile, Liu et al. and Wei et al. have integrated the technology acceptance model with perceived risk theory to predict factors influencing the intention to buy fruits online [56,57]. Likewise, Driediger & Bhatiasevi argue that perceived risk and enjoyment impact perceived usefulness and the intention to use online grocery shopping [58]. In addition, Tandon et al. have integrated the unified theory of acceptance and use technology 2 (UTAUT2) with perceived risk to predict the factors that drive the intention to adopt cash-on-delivery services [59]. Thus, perceived risk influences the intention to use online grocery shopping [60].

In 2020–2022, research trends throughout 2020–2021 discussed the impact of the COVID-19 pandemic on changing consumer shopping behavior [[61], [62], [63]]. Some of these changes in shopping behavior include the tendency to hoard behavior during a pandemic due to anxiety about facing isolation and self-quarantine at home [20,64]. In addition, Piroth et al. stated that personality also contributes to the desire to buy groceries online [65]. Meanwhile, Al Amin et al. and Gumasing et al. argue that consumers tend to choose online grocery shopping during the COVID-19 pandemic due to health consciousness [66,67]. In other words, Dominici et al. stated that sociodemographic and situational factors of the COVID-19 pandemic influenced online grocery shopping decisions [68]. However, apart from the impact of COVID-19 on grocery shopping behavior, other studies have discussed the factors that drive consumer satisfaction and repurchase intentions in e-grocery, including Annaraud & Berezina which mention the importance of food quality, convenience, customer service, and fulfillment of satisfaction and behavioral intentions [69]. Ma et al. added that delivery quality, product in hand, and packaging are vital in increasing customer satisfaction in online shopping services for fresh grocery products; besides that, the corporate image moderate's satisfaction and repurchase intention [70]. In comparison, Jain et al. argue that availability, timeliness, and condition are dimensions of measuring the quality of e-logistics services as a catalyst for satisfaction and repurchase intentions [71]. From a different perspective, Handayani et al. stated that consumer mobility, perceived search effort, and delivery services influence customer intentions to switch from shopping in traditional markets to online grocery shopping [72]. In contrast, Correa et al. and R. Singh & Rosengren argue that switching costs are essential for customers to determine whether they intend to switch, stay, or even continue using online grocery shopping [73,74]. Furthermore, Droogenbroeck & Van Hove developed the UTAUT2 model by adding the possible influence of perceived risk, shopping enjoyment, customer innovation, perceived time pressure, and service quality to predict purchase intentions in e-grocery [26].

### 4.3 Research gaps

**Fig. 5:**
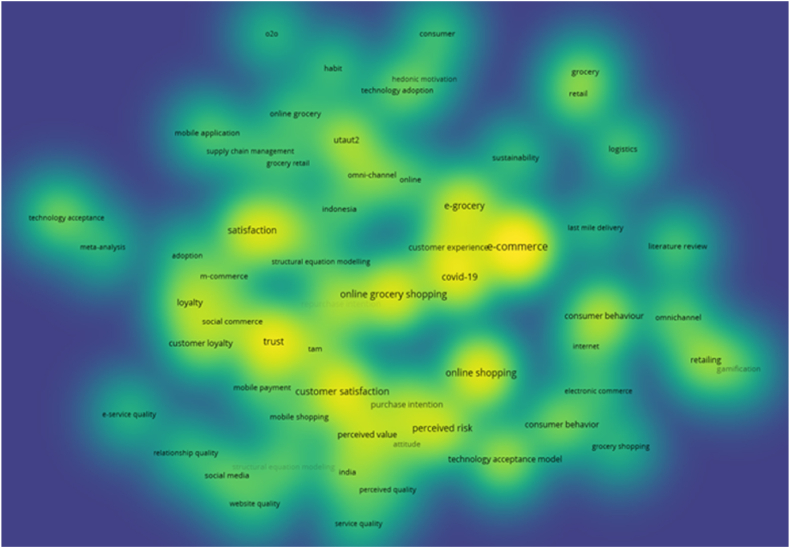
Density visualization of data.

The density visualization in bibliometrics given by VOS Viewer involves depicting the concentration of relationship within a network. The graphical presentation illustrates network areas where entities (keywords) are strongly linked. Higher density in a single region indicates more interactions among the entities. In a bibliometric study, density visualization is especially effective for identifying a research gap which is a topic or feature of a field that has received limited attention or underexplored in the current literature. Fig. 5 present the level of density of research topics in the period 2012 to 2022.

Based on the visualization of density, it can be seen that the density of e-commerce research is higher when compared to other topics with the brightest highlights. The research topics with dimmer highlights and far-reaching relationships between keywords indicate that there is still space for unfilled research gaps and in-depth exploration of topics that can bring up new findings. In particular, the big topic of e-grocery allows for further exploration regarding last-mile delivery, logistics, e-service quality, perceived quality, omnichannel, and relationship quality with research objectives on e-grocery services in Indonesia.

### 4.4 Systematic literature review

**Fig. 6:**
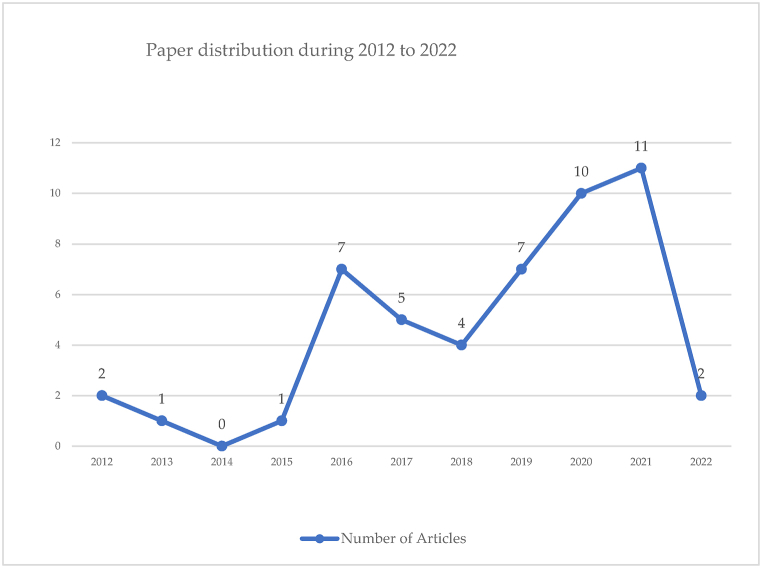
Number of articles published.

Based on the results of a review of the 50 articles selected through several PRISMA diagram stages, the findings show that research in online grocery shopping is moving dynamically from year to year. Fig. 6 illustrates the number of paper distributions on the topic of online grocery shopping that are published each year.

Fig. 6 shows that from 2012 to 2015, research focused more on e-commerce and online shopping, whereas niche topics such as grocery shopping came up less frequently. In 2016, online grocery shopping started to catch the attention of researchers. Despite experiencing a slight decline in 2018, the share of paper publications has increased again in the following years; even in 2021, paper publications related to online grocery shopping occupy the highest paper distribution position.

**Fig. 7:**
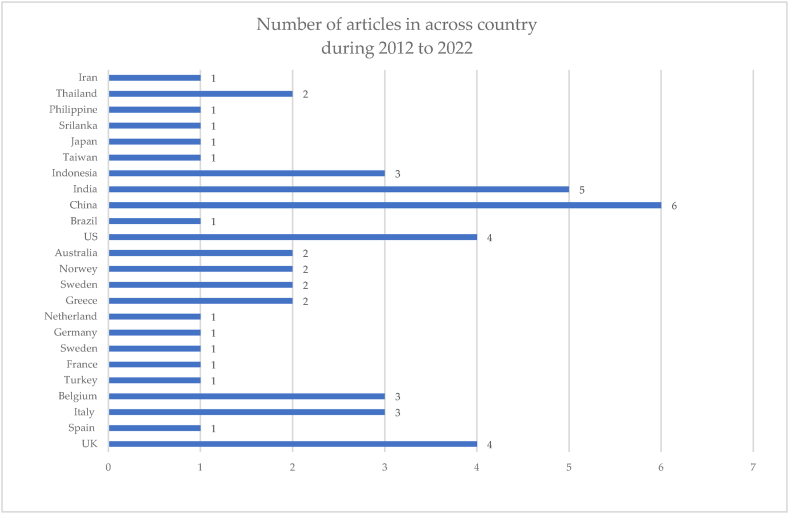
Research of grocery shopping in across country during 2012–2022.

Furthermore, based on the research's location, the literature review results show that research on online grocery shopping has been conducted in 22 countries. Fig. 7 illustrates the distribution of papers by location (country) of case studies. Based on Fig. 7, it can be seen that of the 50 selected articles, there were six articles research cases in China (12%) and this is the highest distribution rate compared to the number of case studies in other countries. Then, there were five cases in India (10%), four cases each in UK and US (8%), three cases each in Belgium, Italy, and Indonesia (6%), two cases each in Greece, Belgium, Norway, Australia, and Thailand (4%), and one case each in Spain, Turkey, France, Sweden, Germany, Netherland, Taiwan, Japan, Philippine, and Iran (2%).

**Fig. 8:**
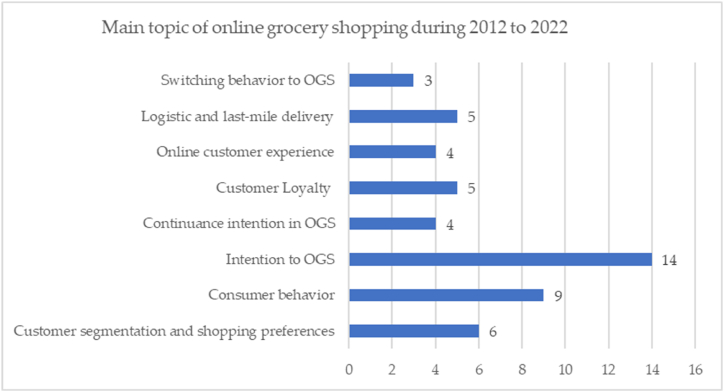
Main topics on grocery shopping during 2012–2022.

Next, Fig. 8 describes the data on the distribution of research topics based on the results of a review of the 50 selected articles.

Based on Fig. 8, it can be seen that research that analyses the factors that drive the intention to use online grocery shopping services is a trending research topic from 2012 to 2022; there are 14 articles reviewed (28%). Many researchers took up this case to further analyze what factors can increase the intention to shop for groceries online. The results of this study certainly help provide solutions and directions for retail companies to design promotional strategies to increase the attractiveness of online grocery shopping services. Furthermore, the researcher also chose many topics about consumer behavior to dig deeper into why someone chooses online grocery shopping services; namely, nine articles have been reviewed (18%). Several studies suggest that socio-demographic characteristics often shape behavior; even social or situational influences often contribute to consumer behavior. Then, the researchers also raised many other main topics, such as customer segmentation and shopping preferences, continuance intention in online grocery shopping, customer loyalty, switching behavior, logistics and last-mile delivery, and the supply chain of the retail grocery business.

### 4.5 Main topics on online grocery shopping

Based on the results of a literature review analysis of the 50 full-text articles presented in Fig. 8, this study found nine main topics in online grocery shopping, namely: customer segmentation and shopping preferences, consumer behavior, intention to online grocery shopping, continuance intention and loyalty, online customer experience, logistics and last-mile delivery, supply chain, and switching behavior. The results of the researcher's review of the paper in detail can be seen in Appendix 1 and the following discussions.

#### 4.5.1 Customer segmentation and shopping preferences

Atkins et al. explained that there are three grocery smart shopper segments, namely spontaneous, apathetic, and involved smart shoppers [41]. Furthermore, through cluster analysis, this study compares the behavior of each customer segment to assess the factors that drive their preference in grocery shopping. These factors include information search, planning for purchases, saving effort, getting the right product, saving money, and saving time. The three customer segments stated that saving effort and saving time are important factors that determine the level of satisfaction and the decision to choose grocery shopping services. Likewise, Chintagunta et al. stated that shopping value influences grocery shopping preferences, whether offline or online [39]. Working consumers with high busyness tend to prefer grocery shopping online to save shopping time. In addition, the availability of delivery services is the main attraction of online shopping services [75]. Interest in online grocery shopping has increased during the COVID-19 pandemic because people avoided physical shopping transactions that endangered their health [76,77]. However, unlike Maruyama et al. which state that consumers still prefer to shop at physical stores compared to online stores to get fresher products [46]. In line with Saphores & Xu who stated that consumers generally prefer to shop physically [63] – because women who have a high level of household chores and have no access to grocery stores will prefer online shopping.

Research on customer segmentation used methods such as cluster analysis, conjoint analysis, and regression analysis to analyze the relationship between socio-economic characteristics of customers on online grocery shopping decisions. While the topic regarding shopping preferences on average uses econometric modeling, probit models, logit models, one-way manova, and the Bayesian information criteria to analyze the effect of the shopping experience, perceived product quality, perceived price, and delivery service on customer preferences in choosing shopping services grocery.

#### 4.5.2 Consumer behavior on online grocery shopping

Conlin & Labban stated that demographic characteristics influence the decision to determine shopping services [78]. It is proven that household characteristics play a role in spending decisions. Online grocery shopping customers are generally young families with small children. Likewise, Dhaoui et al. ague that socio-economic factors, which include age, education level, and income level play a role in determining grocery shopping channels [79]. In addition, Janssen et al. stated that customer value of the quality of products and services also influences grocery shopping behavior [80]. In more depth, Berg & Henriksson explained that good financial support is needed (remembering that online shopping is more expensive than physical shopping), flexibility in receiving product shipments, and sound technology facilities at home to ultimately prefer grocery shopping online rather than physical shopping [81]. It can be said that situational factors greatly influence online grocery shopping decisions [82] as Hao et al. and Hartono et al. stated that interest in online grocery shopping increased rapidly during the COVID-19 pandemic due to consumers' limited access to physical stores and various health reasons [20,64].

Research on online grocery shopping behavior has used descriptive analysis, logit model, and manova analysis methods to explain the influence of socio-economic characteristics of customers' households on the behavior of online grocery shopping services. This research was the main topic during the COVID-19 pandemic because researchers wanted to find out how much influence factors of purchasing power, working hours, grocery store accessibility, technology literacy, situational factors, and health consciousness had on grocery shopping behavior at that time.

#### 4.5.3 Intention to online grocery shopping

From 2012 to 2022, the topic of online grocery shopping research discussed a lot about the factors driving the intention to use online grocery shopping services. Zhu & Semeijn stated that flexibility in ordering time and fast delivery services are the main attractions of online grocery shopping [36]. Likewise, Zhao et al. stated that the fulfillment of delivery services is an essential factor influencing customer intentions to shop online for fresh grocery products [83]. Next, Alavion et al. used a theory of planned behavior (TPB) approach to predict consumer behavior toward digital marketing of fresh agricultural products [44]. Meanwhile, Shukla & Sharma used the technology acceptance model (TAM) approach to predict the intention to use online grocery shopping services [84]. On the TAM expansion, Driediger & Bhatiasevi, Jun et al. and Loketkrawee & Bhatiasevi explained that the convenience, benefits, and convenience of services are significant factors driving the intention to use online shopping services [55,58,85]. In line with Anesbury et al. who explained that the ease of use of technology is vital to encourage consumer interest in using online grocery shopping services [43].

Furthermore, Chakraborty [86] has integrated TPB and TAM to predict intentions to use grocery shopping applications [87]. More broadly, Droogenbroeck & Van Hove used the unified theory of acceptance and use technology 2 (UTAUT2) approach to look at the factors that influence consumers to adopt e-grocery shopping services [27]. During the COVID-19 pandemic, Gumasing et al. stated that the interest in online grocery shopping is due to perceived benefits, performance expectancy, and cues to action [67]. Research on intention to use online grocery shopping generally uses the structural equation modeling method to explain the factors driving purchase intention.

#### 4.5.4 Continuance intention to online grocery shopping

Fresh grocery products have perishable characteristics, so customer perceptions of the risk and satisfaction of shopping online for grocery products contribute to growing customer trust, encouraging repurchase intentions [88]. P. Singh et al. added that apart from perceived risk, online tracking, online rating, review, and design aesthetics of a website also play a role in encouraging satisfaction and intention to reuse e-grocery shopping services [89]. Furthermore, Asti et al. explained that price fairness influences perceived value which encourages repurchase intentions, and service excellence plays a role in increasing satisfaction which encourages customer trust and repurchase intention [90]. In general, research to predict the factors that drive continuance intention to use online grocery shopping uses the structural equation modeling analysis method and determines which factors give the most impetus to continuance intention.

#### 4.5.5 Customer loyalty

Service quality drives customer satisfaction and loyalty to grocery stores [3]. Service quality measurement includes physical aspects, reliability, personal interaction, and policies. Then, Sivapalan & Jebarajakirthy added that the information conveyed by retailers regarding physical aspects, reliability, personal interaction, problem-solving, and procedures is the driving force behind the growth of customer loyalty [91]. In contrast, Moriuchi & Takahashi used a marketing mix concept approach that includes product quality, price, promotion, and place, as well as a pleasant shopping experience to encourage satisfaction and trust, which can foster customer loyalty using online shopping services at supermarkets [92]. Maggioni explored customer perceptions of grocery shopping services through associative network analytics, mentioning several essential factors supporting the continuity of the retail grocery business, namely value for money, convenience, ease of getting benefits, product availability and diversity, product quality (freshness), and customer relationship services [48]. Furthermore, Sreeram et al. used the TAM approach and its expansion to predict the driving factors of e-grocery customer loyalty [93] and stated that perceived usefulness and social influence were essential factors influencing loyalty.

#### 4.5.6 Online customer experience

Campo & Breugelmans stated that the difference in consumer experience when using offline and online services determines the customer's decision to choose service channels in the future [94]. Singh and Singh & Söderlund stated that customer service, website quality, product quality, delivery services, and brand experience contribute to the online grocery shopping experience, which in turn increases satisfaction and has the potential to increase repurchase intention and word-of-mouth (WOM) [95,96]. The same thing was conveyed by Vakulenko et al. that online experience directly increases customer satisfaction using online retail services [54]. However, last-mile delivery also mediates between the online experience and satisfaction.

Research on online customer experience with a quantitative approach uses the structural equation modeling analysis method. In contrast, in a qualitative approach, Singh used exploratory and netnographic analysis to explain the influence of personal and psychological characteristics of consumers on hedonic and utilitarian shopping values, which are then reflected in the impression consumers and online customer experience when using online grocery shopping services [96].

#### 4.5.7 Logistic service and last-mile delivery

Kaswengi & Lambey-Checchin viewed the importance of logistics services to support product quality (product freshness) as an essential factor that drives consumer satisfaction and increases the volume and frequency of online grocery shopping [53]. Furthermore, de Kervenoael et al. explained that the quality of logistics services, which include retail infrastructure, time management, logistics operational procedures, and service convenience, are the attractions of online grocery shopping [97]. In comparison, Jiang et al. argued that the quality of personnel service, delivery service, and timeliness of delivery are the most significant factors contributing to the quality of logistics services in increasing customer satisfaction [98]. However, the price of services is also an influential factor in supporting service quality [99]. Even Cagliano et al. saw that the quality of logistics services needs to be supported by the quality of real-time and integrated information to support order management, transaction management, and fulfillment of delivery services [100]. Research on logistics service and last-mile delivery generally uses structural equation modeling and econometric modeling to predict the potential of logistics service quality or last-mile delivery factors on customer satisfaction when shopping for groceries online. In addition, research on last-mile delivery also uses the system dynamics analysis method to measure the competitiveness of online shopping services compared to physical shopping services and other online shopping services.

#### 4.5.8 Switching intention to online grocery shopping

Through a push-pull-mooring framework, R. Singh & Rosengren explained that the delivery service factor is an attraction for switching to online grocery shopping services [74]. Meanwhile, switching costs are a mooring factor that can hinder or support service transfers, such as the ease of learning electronic services and relatively affordable service costs. Handayani et al. stated that the price factor, delivery time, level of mobility, and perceived channel risk drive the transfer of shopping intentions from traditional markets to online grocery shopping [101]. Next, T. H. N. Nguyen et al. added that price, product quality, and health consciousness were push-pull-mooring factors influencing the intention to switch to online grocery shopping services during the COVID-19 pandemic [102]. Generally, research on switching intention uses the structural equation modeling analysis method to predict potential push, mooring, and pull factors for switching intentions to online grocery shopping services.

## 5 Conclusions, limitations, and future direction

This study analyzed 571 articles on grocery shopping from 2012 to 2022, revealing a predominant research trend focusing on predicting the intention to use online grocery shopping through technology acceptance models. Shifts in emphasis occurred from 2015 to 2016, addressing continuity in grocery shopping services, and from 2017 to 2018, exploring factors influencing shopping intentions, including model extension and integration. The expansion of the technology acceptance model has involved the variables perceived risk, price, service quality, service delivery, and perceived value to predict repurchase intention. Even the Unified Theory of Acceptance and Use Technology (UTAUT) has become a research trend from 2017 to 2018. Meanwhile, from 2019 to 2022, many studies have discussed the influence of the COVID-19 pandemic on changes in grocery shopping behavior. Logistics and last-mile delivery are becoming a trend because delivery services support online grocery shopping services.

The study's contribution lies in identifying research gaps in last-mile delivery, logistics, e-service quality, perceived quality, omnichannel, and relationship quality. It emphasizes the need for further exploration in these areas. For the fresh products recognized as perishable, the study underscores the importance of maintaining product quality during home delivery, highlighting last-mile delivery and logistics services as critical areas. The effectiveness of omnichannel services is deemed essential for improving overall service quality and convenience. Enhancing customer relationship quality is a potential avenue for broader business continuity.

Based on the findings, the managerial implication suggests managers in the online grocery shopping industry can refine the company strategies. The literature study acknowledged the rising demand since 2016 and focused on recommendations to enhance last-mile delivery and logistics services for perishable products. The effectiveness of omnichannel services should be a key consideration for improving overall service quality and convenience. Emphasizing and improving customer relationship quality is a potential avenue for enhancing overall service quality and ensuring business continuity.

The study acknowledges limitations, analyzing 571 papers and reviewing 50 articles from 2012 to 2022, exclusively in English-language papers from Scopus-accredited journals with open-access status. The dynamic nature of published papers and potential omissions during manual reviews highlight the need for more comprehensive data collection to thoroughly analyze topic trends and research gaps in online grocery shopping. The study suggests a pressing need for further research to collect more data and conduct a more extensive analysis of topic trends and research gaps in online grocery shopping. Future studies should overcome identified limitations, considering a broader range of publications, languages, and sources to ensure a comprehensive understanding of the evolving landscape. Exploring emerging technologies, consumer behaviors, and external factors influencing the online grocery shopping industry can contribute valuable insights for academics and industry practitioners.

## CRediT authorship contribution statement

**Terrylina A. Monoarfa:** Writing – original draft, Visualization, Methodology, Formal analysis, Conceptualization. **Ujang Sumarwan:** W. **Arif Imam Suroso:** Writing – review & editing, Supervision. **Ririn Wulandari:** Writing – review & editing, Supervision.

## Declaration of competing interest

The authors declare that they have no known competing financial interests or personal relationships that could have appeared to influence the work reported in this paper.

## Figures and Tables

**Table:** 

Topic	Factors	Methods	Findings	References
Customer segmentation	Customer socio-economics characteristics, customer involvement, perceived saving effort and time, perceived price, product quality, and service quality, attitude, situational factors, health consciousness	Cluster analysis, conjoint analysis, regression analysis	Retailers need to increase the convenience of the shopping experience to engage customer segments. Basically, saving effort and saving time is key factors to increase customer satisfaction. For high-income women, they tend to buy groceries online in small quantities, but with high frequency. However, for elderly customers, mobility, distance to the nearest grocery store, and health condition are considerations when choosing to shop for groceries online.	[41,78,82,83]
Shopping preferences	Product quality (freshness, safety), price, service quality, cost (transaction, transport), lead time (time window), promotion, online experience, socio-economics characteristics	Econometric modeling, probit model, logit model, one-way manova, the bayesian information criteria.	Customers prefer to shop offline for sensory products and perishable products. Meanwhile, for large-sized products, customers prefer to shop online to fulfill a comfortable shopping experience. The process of waiting for delivery of orders often becomes inefficient, thus encouraging customers to prefer conventional shopping. To grow online shopping intentions, retailers need to determine the right price and time window strategy, not only to meet customer expectations, but also to maintain product quality. However, among several logistics services, it turns out that delivery services are the most popular during the Covid-19 pandemic.	[39,46,76,77,79,94]
Consumer behavior	Socio-economics characteristics, price, service quality, logistic service, situational factors, health consciousness, attitude	Descriptive analysis, logit model, manova analysis	Household socio-economic characteristics play a vital role in determining the use of online grocery shopping services. Purchasing power, working hours, perceived availability and technology literacy are some of the reasons that drive consumers to prefer shopping for groceries online. In times of a pandemic, situational factors and health awareness are the biggest drivers of intentions to adopt online grocery shopping. However, research findings indicate that there is an acute problem in the delivery of perishable foodstuffs due to long distances that require a certain amount of time.	[20,63,64,81]
Logistic and last-mile delivery	Product quality (freshness), time-based delivery (timeliness), cost of delivery, logistic operational procedure, promotion, convenience	Structural equation modeling, econometric modeling, system dynamics	Basically, customers are focused on product fulfillment and lead time delivery. In addition, real time information will facilitate payment transactions and logistics management that impact mobile application performance. In particular, customers expect logistics services that include personal contact, flexibility in choice of delivery time and range, and empathy to enhance shopping convenience and satisfaction.	[53,75,97,98,100] [53,75,97,98,100]
Online customer experience	Experience of task (service and customer ROI), pleasure, website, product, brand, last-mile delivery	Structural equation modeling, netnography	Customer service, delivery service, and brand experience are the main factors that enhance online grocery shopping experience and satisfaction which ultimately drive repurchase intention. Customers expect retailers to provide services efficiently for the investment paid. The difference in the amount of ROI has the potential to encourage the intention to switch.	[54,95,96]
Intention to use online grocery shopping	Perceived ease of use and usefulness, product quality, service quality, price, time-saving, shipping time, lead time delivery, perceived risk, enjoyment, website, facilitating condition, social influence, personal innovativeness, self-efficacy, trust, customer characteristics	Descriptive analysis, regression analysis, structural equation modeling, binary logit choice model	The user's technological skills and facilitation conditions are very important to encourage the intention to adopt e-grocery applications. In addition, the ease of use is the main attraction for new users. Furthermore, perceived benefits, enjoyment, and trust contribute to behavioral intentions to use e-grocery applications. The Covid-19 pandemic period brought new habits for consumers to use e-grocery shopping services and home delivery services, which became one of the most popular features for customers.	[27,36,43,44,55,58,67,83,84,99,103] [27,36,43,55,58,67,83,84,99,104]
Continuance intention to use online grocery shopping	Online shopping experience, online tracking, online review and rating, product quality, service quality, price, perceived value, perceived risk, satisfaction, trust	Structural equation modeling	Perceived price, product quality, service quality, perceived risk, and perceived value are drivers on repurchase intention. For infrequent online grocery shoppers, perceived risk and shopping experience are important factors to increase trust and repurchase intention.	[49,80,89,90]
Customer loyalty	Marketing mix aspects, perceived ease of use and usefulness, service quality, time delivery, mobility, social influence, online experience, customer characteristics	Structural equation modeling	Service quality is essential to maintain the customer loyalty and improve competitive advantage of grocery store. However, for online grocery shopping, the environmental stimuli (such as aesthetic design and user-friendly navigation) enhance the convenience and customer loyalty.	[3,47,91,93]
Switching intention	Customer service, product quality, delivery service, price, perceived risk, WOM, health consciousness, switching cost, customer characteristics	Structural equation modeling	Perceived risk, price, and time delivery are the customers' consideration to use online grocery shopping. However, switching costs, willingness to pay, and alternative attractiveness are the main key to switch to online grocery shopping.	[72,74,102]
